# A semi-experimental procedure for the estimation of permeability of microfluidic pore network

**DOI:** 10.1016/j.mex.2019.03.025

**Published:** 2019-04-02

**Authors:** Sushobhan Pradhan, Imran Shaik, Rudy Lagraauw, Prem Bikkina

**Affiliations:** a Oklahoma State University, Stillwater, OK, USA; b Micronit Microtechnologies, Enschede, Netherlands

**Keywords:** Semi-experimental procedure for the estimation of permeability, Microfluidics, Permeability, Pressure drop, Hydraulic resistance, Darcy’s law, Kozeny-Carman

## Abstract

Microfluidic porous media systems are used for various applications ranging from chemical molecule detection to enhanced oil recovery studies. Absolute permeability data of the microfluidic porous media are important for those applications. However, it is a significant challenge to measure the permeability due to the difficulty in accurately measuring the ultra-low pressure drop across the pore network. This article presents a semi-experimental procedure to estimate the permeability of a microfluidic pore network. The total pressure drop across the porous media chip (ΔP_chip_) at a given flow rate of a single-phase liquid was obtained from the difference in the inlet pressures at the microfluidic pump with and without the pore network chip connected. The pressure drops in the inlet (ΔP_inlet channel_) and outlet (ΔP_outlet channel_) channels of the pore network are estimated using the hydraulic resistance equation for Poiseuille flow in a wide rectangular cross section. Then the pressure drop across the pore network of the chip (ΔP_pore network_) is obtained by subtracting (ΔP_inlet channel_ + ΔP_outlet channel_) from ΔP_chip_. Subsequently the permeability of the pore network is calculated using the Darcy’s law.

• The proposed method is applicable for both homogenous and heterogeneous pore networks.
• This method does not require a differential pressure sensor across the microfluidic chip.
• This method eliminates the possibility of gas entrapment that can affect the permeability measurement.

## Graphical abstract

**Figure:**
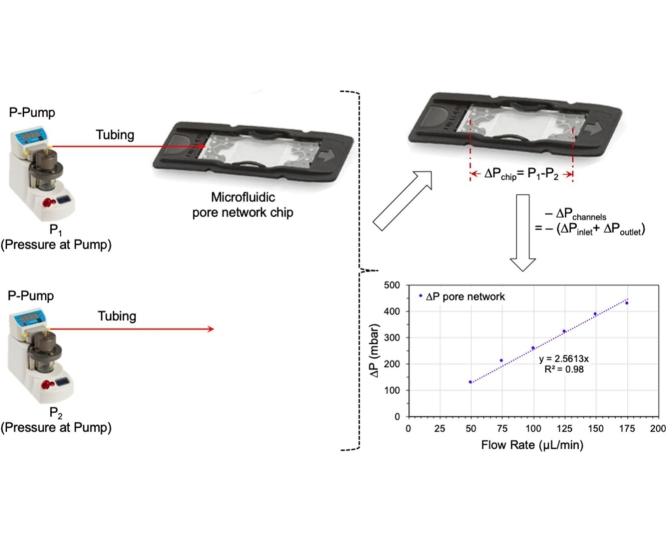

**Specifications Table**

**Table:** 

Subject Area:	Engineering
More specific subject area:	Microfluidics; Porous media flow
Method name:	Semi-experimental procedure for the estimation of permeability
Name and reference of original method:	Not applicable
Resource availability:	Not applicable

## Method details

Microfluidics has become an important tool in various process and analytical technologies [[1], [2], [3], [4], [5], [6], [7], [8]]. Microfluidic porous media systems are used for chemical molecule detection, preconcentration of proteins, purification of bio-macromolecules, 3D cell culture, mixing-controlled reactions, and enhanced oil recovery studies [[9], [10], [11], [12], [13], [14], [15], [16], [17], [18]]. Permeability is a property of the porous media and is independent of the liquid flows through it. However, the liquid must be a single phase and inert to the solid. Permeability indicates the ability of the porous media to allow a fluid to flow through it. Permeability measurement of microfluidic porous media is important for many applications. For example, relative permeability curves are necessary for understanding the multiphase fluid flow behavior in subsurface porous media and reservoir simulation. The relative permeability curves require absolute permeability of the porous media. However, it is a significant challenge to measure the permeability due to the difficulty in accurately measuring the ultra-low pressure drop in the porous media and also in avoiding air entrapment in the porous media and/or flow lines.

Darcy’s law is used to calculate the permeability of a porous media. It states that the rate of fluid flow is directly proportional to the hydraulic gradient (i.e., pressure drop per unit length) and cross-sectional area, and inversely proportional to the viscosity of the fluid. The proportionality constant is the permeability of the porous media. The Darcy’s law for one-dimensional horizontal linear system is defined as follows:

(1) $$q=\;-\frac{kA}{\text{μ}}\frac{\left(P_{1}-P_{2}\right)}{L}$$

where, q is the volumetric flow rate of the fluid in cm^3^/s, k is the absolute permeability in Darcy, A is the cross-sectional area of the porous media in cm^2^, $\frac{\left(P_{1}-P_{2}\right)}{L}$ is the pressure gradient in atm/cm, and μ is the fluid viscosity in cP. The inherent problem in measuring the permeability of a microfluidic porous media is in accurately measuring the ultra-low pressure drop which is usually in the order of millibars. Air entrapment is another significant problem especially when the differential pressure transducer across the microfluidic chip or the absolute pressure transducer at the inlet of the chip is perpendicularly connected to the main flow line of the microfluidic chip.

Joseph et al. measured the permeability of four different types of pore networks by measuring the absolute pressure near the inlet of the microfluidic chip [19]. The outlet tubing of the chip was open to atmosphere. The pressure drops in the inlet and outlet tubing and the inlet and outlet sections of the pore network in the chip were estimated using Hagen-Poiseuille equation. The differential pressures required to calculate the permeability of the pore networks were calculated by subtracting the sum of the above pressure drops from the corresponding inlet pressures. Since the actual pressure drop in the chip is very low, any error in the estimation of the pressure drops in the tubing and the inlet and outlet sections of the porous area would result in a significant error in the calculation of the permeability. Moreover, the chances for air entrapment are higher since the pressure transducer was perpendicularly connected to the main flow tubing and the tubing connected to the transducer is a dead-end. Therefore, in this work, we propose a method where there is no requirement for a pressure transducer that needs to be perpendicularly connected to the main flow line and as well as no requirement for the estimation of pressure drop in the tubing connected to the microfluidic chip. In fact, the proposed method does not need a separate differential or absolute pressure transducer as the pressure is measured at and by the injection pump.

## Materials

A 2 cm × 1 cm uniform pore network microfluidic chip obtained from Micronit Microtechnologies (Enschede, Netherlands) was used in this work. The chip is hydrophilic and has a porosity of 0.52.

**Fig. 1:**
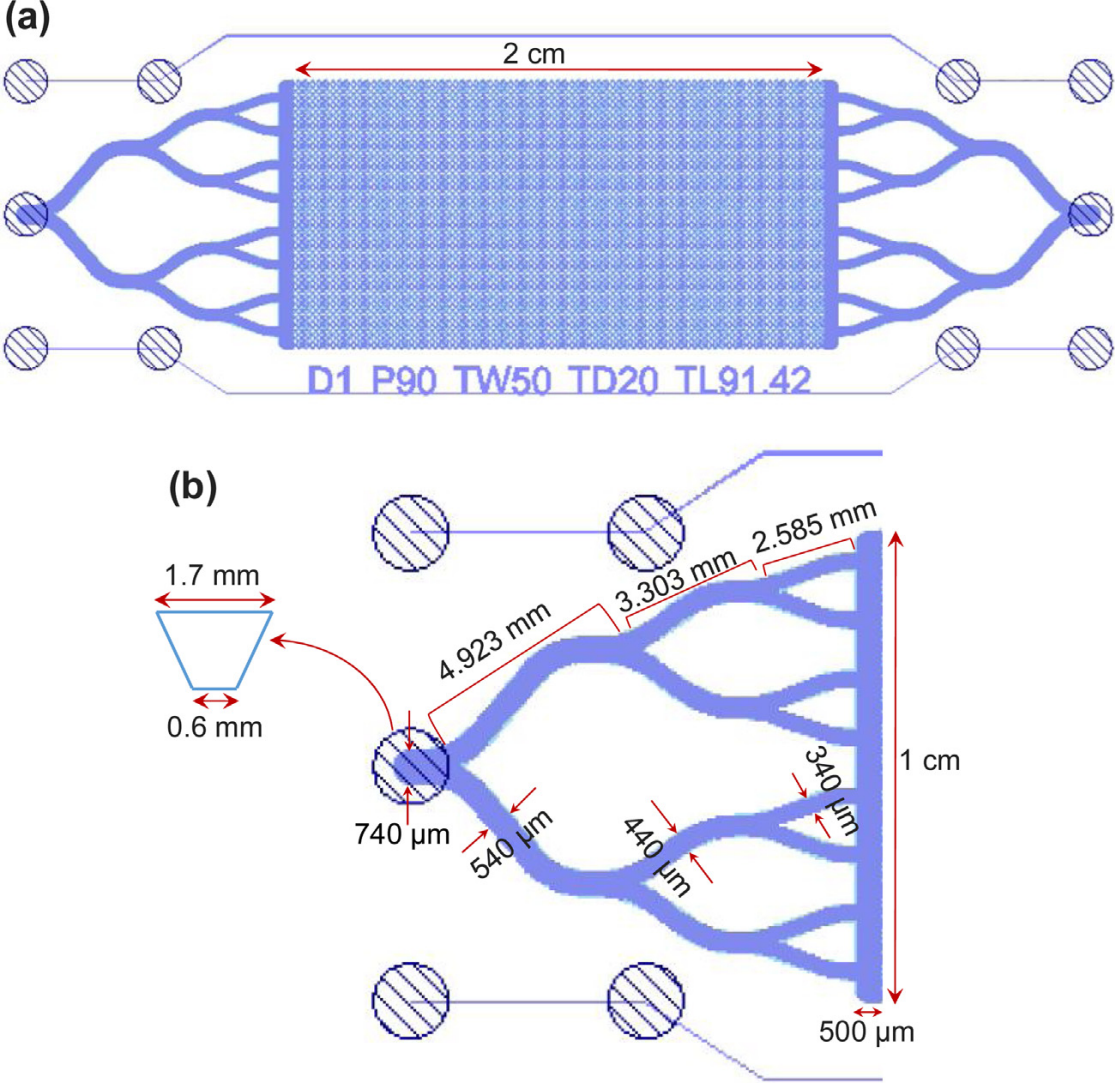
(a) Uniform pore network chip (Notation: D: Design; P: Pore width in μm; TW: Throat Width in μm; TD: Throat Depth in μm; TL: Throat Length in μm); (b) Zoom-in view of inlet port and channels with the dimensions.

The Fig. 1(a) shows a schematic diagram of the microfluidic chip and the Fig. 1(b) shows the zoom-in view of the inlet port and channels, and the corresponding dimensions.

**Fig. 2:**
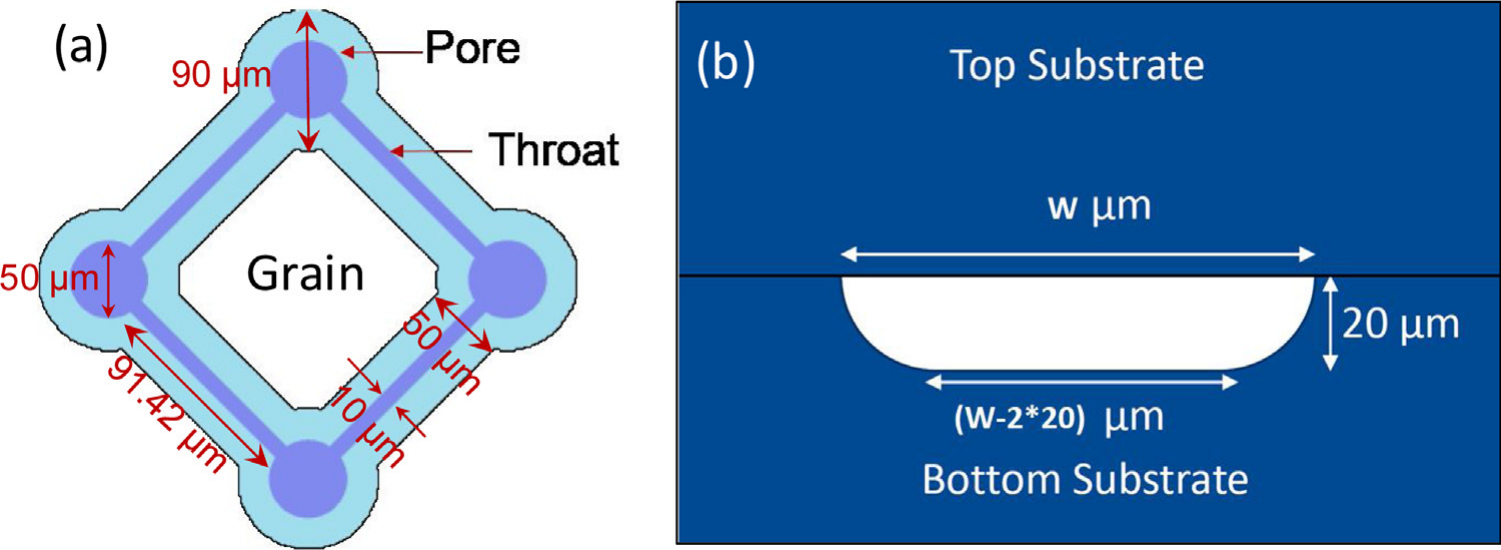
(a) Connection pattern of the pores and throats in the uniform pore network microfluidic chip; (b) Cross-sectional view of a pore or a throat in the microfluidic chip, where ‘W’ is the width.

Fig. 2(a) shows the cross-sectional view of a pore or a throat in the microfluidic chip used in this study. The widths (W) of the pores and throats are 90 μm and 50 μm, respectively. The depth of the pores and throats is 20 μm. The length of the throat is 91.42 μm. Fig. 2(b) shows the connection pattern of the pores and throats. The inlet of the microfluidic chip is a conical hole with a 1.7 mm diameter at the top and 0.6 mm at the bottom and the bottom side is connected to a channel of 740 μm width. The channel is successively bifurcated into 540 μm width, followed by 440 μm and 340 μm width channels in the sequence. The corresponding lengths of the three bifurcated channels are 4.928, 3.303 and 2.585 mm, respectively. The eight 340 μm width channels are joined to a vertical channel of 500 μm width and 1 cm height. The vertical channel is connected to the pore network of the microfluidic chip. The shapes of channel cross-sections are similar to that of the pores and throats as shown in Fig. 2(a). The outlet side of the chip is connected to an exactly similar vertical and bifurcated channel structure but in the reverse order. These inlet side and outlet side channel structures allow the fluid to flow through the entire cross section of the pore network.

Deionized (DI) water was used for the permeability measurement. It should be noted that usage of a liquid that can completely wet the solid matrix is critical for permeability measurement as a non-wetting fluid may not be able to displace air from the smaller pores in a non-uniform pore network. We also suggest vacuum saturation of the pore network with the test fluid to ensure no air entrapment.

## Experimental facility

**Fig. 3:**
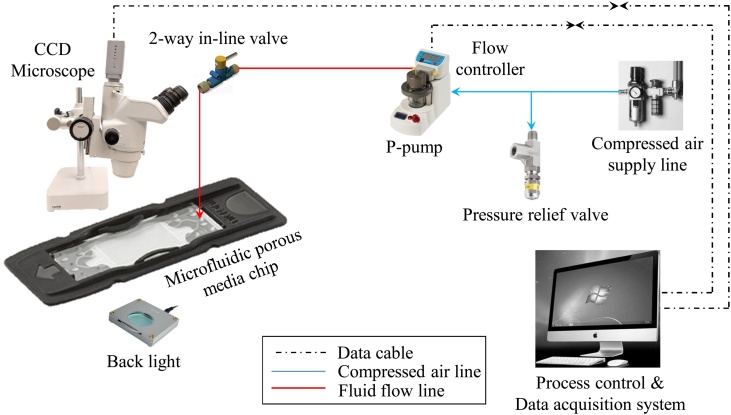
Schematic of the experimental facility used for permeability measurement.

The schematic of the microfluidics facility used for permeability measurement is shown in Fig. 3. The facility consists of a microfluidic pump (Make: Dolomite; Model: 1600933) which is connected to a compressed air supply of 110–130 psig through a pressure relief valve (PRV) set at 125 psig. The compressed air helps to obtain pulseless flow of the test liquid, i.e., DI water in this case. The pump can control the liquid flow rate in the range of 30–1000 μL/min with a resolution of 1 μL/min or pressure in the range of 0–10,000 mbar with a resolution of 1 mbar. The outlet of the microfluidic pump was connected to the inlet of the pore network chip using a 1/16 inch O.D. and 800 μm I.D. tubing, via a flow controller and a 2-way in-line valve. The outlet of the microfluidic chip was open to atmosphere, i.e. no outlet tubing was connected. A microscope (Make: Nikon; Model: SMZ745T) integrated with a high-speed CCD camera (Make: PixeLINK; Model: B742) was used to observe the microfluidic chip to make sure it was free of any trapped air during the permeability measurement.

## Experimental procedure

The following steps were used to estimate the absolute permeability of the pore network of the microfluidic chip:

1 First, DI water was injected at a sufficiently high flow rate to remove all the air from the flow line and the pore network chip. Usually about 500 μL/min DI water flow rate was sufficient to completely displace the air from the chip.
2 Then the flow was adjusted to a test flow rate and the injection pressure (P_1_) was measured at the microfluidic pump while the chip outlet was open to atmosphere. It should be noted that the average pressure of a minimum of fifty readings after the flow stabilization were considered for permeability calculation. This procedure was repeated for other flow rates used in this study (50, 75, 100, 125, 150 and 175 μL/min).
3 After step 2, the chip was disconnected from the inlet tubing and the injection pressures (P_2_) were measured for the flow rates used in step 2.
4 The difference in the two injection pressures provides the pressure drop in the chip (ΔP_chip_) for the corresponding flow rate.
5 The pressure drops in the inlet (ΔP_inlet channel_) and outlet (ΔP_outlet channel_) channels of the pore network were estimated assuming the hydraulic resistance equation for Poiseuille flow in a wide rectangular cross section. This assumption is appropriate as the width >> height (width to height ratios are 27, 22 and 17) of the channels. For a rectangular micro channel, the hydraulic resistance is expressed as follows [20]:

(2) $$R_{h}\approx \;\frac{12\text{μ}L\;}{wh^{3}(1-\frac{0.63h}{w})}$$

where, L, w, and h are the length, width, and height of the channel in m, μ is the fluid viscosity in Pa s, and $R_{h}$ is the hydraulic resistance in Pa s/m^3^.

6 Then the pressure drop in the pore network (ΔP_pore network_) was obtained by subtracting the pressure drop in the inlet and outlet channels (**Δ**P_channels_) from the total pressure drop across the chip.
7 The ΔP_pore network_ was used along with the fluid and pore network properties required to calculate the absolute permeability using the Darcy’s law, i.e., Eq. (1).

## Results and discussion

The hydraulic resistances of the bifurcated channels are estimated using the Eq. (2). The estimated hydraulic resistance data of each bifurcated channel are given in Table 1. The pressure drop across the channels are calculated by multiplying the resistance and the corresponding flow rate in the channel. It is assumed that the flow rate becomes half when each main channel is bifurcated into two equal sized channels, and the slight curved nature of the channels causes insignificant pressure drop [21].

**Table 1:** Estimated hydraulic resistances of inlet and outlet bifurcated channels of the chip.

	Length (L)	Width (w)	R_h_
	m	m	Pa s/m^3^
1st Bifurcation	4.928 × 10^−3^	5.4 × 10^−4^	1.31 × 10^13^
2nd Bifurcation	3.303 × 10^−3^	4.4 × 10^−4^	1.08 × 10^13^
3rd Bifurcation	2.585 × 10^−3^	3.4 × 10^−4^	1.10 × 10^13^
Entrance channel	5 × 10^−4^	1 × 10^−2^	6.99 × 10^10^

The measured pressure drops across the chip, and the estimated pressure drops across the channels, and pore network for the flow rates used in this study are summarized in Table 2.

**Table 2:** Summary of the measured and estimated pressure drop data at different flow rates.

Flow Rate (μL/min)	P_1_ (mbar)	P_2_ (mbar)	ΔP_chip_ (mbar)	ΔP_channels_ (mbar) (from Eq. (2))	ΔP_pore network_ (mbar) (ΔP_chip_ - ΔP_channels_)
50	2176 ± 2	1870 ± 4	306	178	128
75	3301 ± 7	2823 ± 9	478	267	211
100	4410 ± 21	3795 ± 9	615	356	259
125	5496 ± 41	4730 ± 7	766	445	321
150	6628 ± 9	5706 ± 9	922	534	388
175	7668 ± 30	6616 ± 15	1052	623	429

**Fig. 4:**
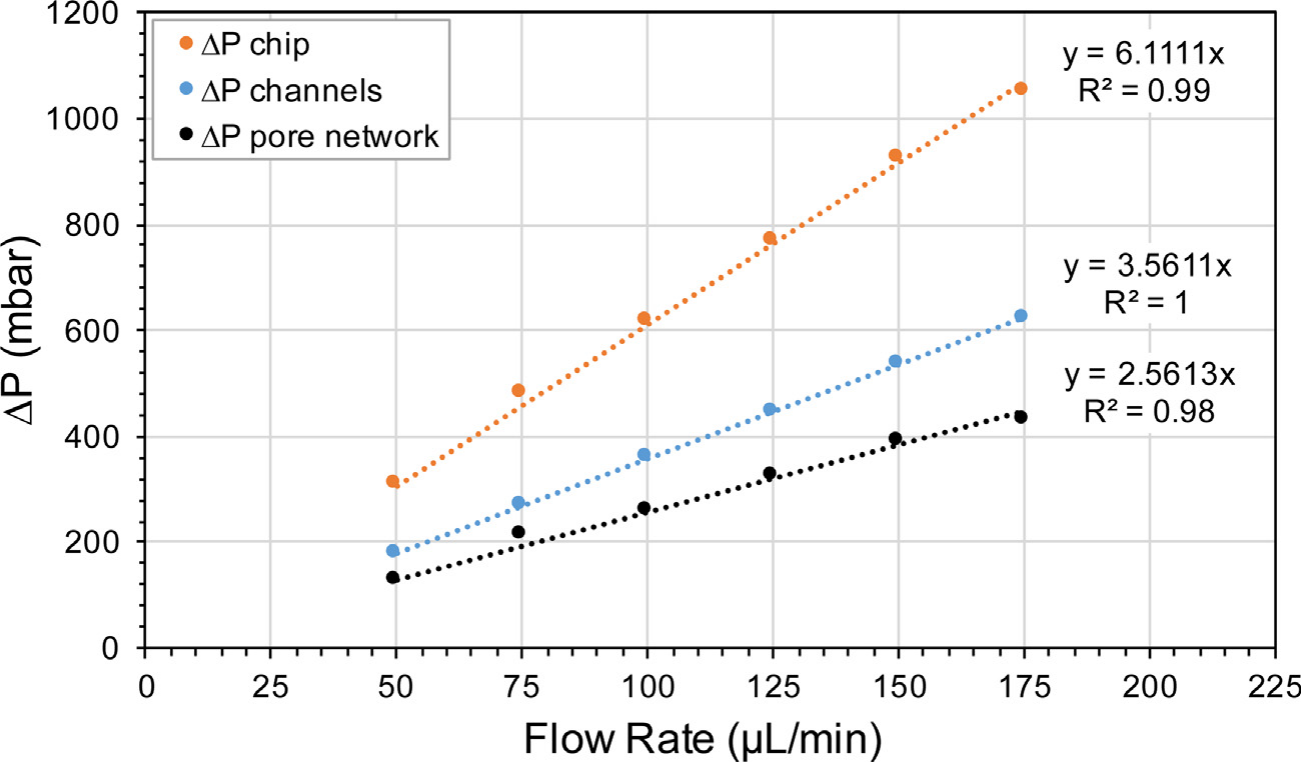
Pressure drops across chip, channels, and pore network at different flow rates.

The Fig. 4 shows the trends of flow rate versus the pressure drop across the chip, channels, and the pore network. It should be noted that the pressure drop in the inlet and outlet channels are very significant compared to the pressure drop in the pore network and hence the pressure drops in the inlet and outlet channels cannot be ignored for permeability estimation.

### Procedure for permeability estimation

The procedure followed to calculate the permeability of the pore network is given below.

Volumetric flow rate (q) = 100 μL/min = 0.00167 cm^3^/s

Viscosity (μ) of water at 23 °C = 0.9321 cp

Length of the pore network = 1 cm

Depth of the pore network = 20 μm = 0.002 cm

Cross-sectional area (A) of the pore network = 0.002 cm^2^

P_1_ (measured at the P-Pump) = 4410 mbar

P_2_ (measured at the P-Pump) = 3795 mbar

ΔP_chip_ = 4410 – 3795 = 615 mbar

ΔP_channels_ = ΔP_inlet_ + ΔP_outlet_ = 2 × ΔP_inlet_ = 356 mbar

ΔP_pore network_ = ΔP_chipSc_ – ΔP_channel_ = 615 – 356 = 259 mbar = 0.256 atm

Therefore, from the Darcy’s law given in the Eq. (1), the permeability of the pore network, k = 6.39 Darcy

The same procedure is followed to estimate the pore network permeability for the other flow rates. The permeability data are summarized in Table 3. As expected, within the Darcy’s flow regime, the absolute permeability is practically a constant and independent of the flow rate.

**Table 3:** Estimated permeabilities at various flow rates.

Flow Rate (μL/min)	Permeability (Darcy)
50	6.47
75	5.89
100	6.39
125	6.45
150	6.40
175	6.75

From Table 3, average permeability, k_avg_ = 6.39 ± 0.28 Darcy.

### Comparison of the semi-experimental and Kozeny-Carman permeabilities

The estimated permeability of porous media chip using the semi-experimental procedure is compared with the permeability estimated using the Kozeny-Carman equation given below [22]:

(3) $$k=\;\frac{{\text{∅}}_{s}^{2}D_{g}^{2}}{150}\frac{\varepsilon^{2}}{1-\;\varepsilon^{2}}$$

where, $\emptyset_{s}$ is the sphericity of the grain in the microfluidic chip, $D_{g}$ is grain diameter, and ε is porosity of the microfluidic chip.

Porosity of uniform network chip, ε = 0.52

Surface area of the grain (shown in Fig. 2(a)), S_g_ ≈ 32742 μm^2^

Volume of the grain, V_g_ ≈ 240722 μm^3^

Grain diameter, *D*_g_ = ${\left(\frac{6\;v_{g}}{\pi }\right)}^{1/3}$ ≈ 77.2 μm

Sphericity = 6DgSgVg ≈ 0.57

Therefore, k (from the Kozeny-Carman equation) ≈ 4.81 μm^2^ ≈ 4.87 Darcy

k (using the semi-experimental procedure) = 6.39 Darcy

**Fig. 5:**
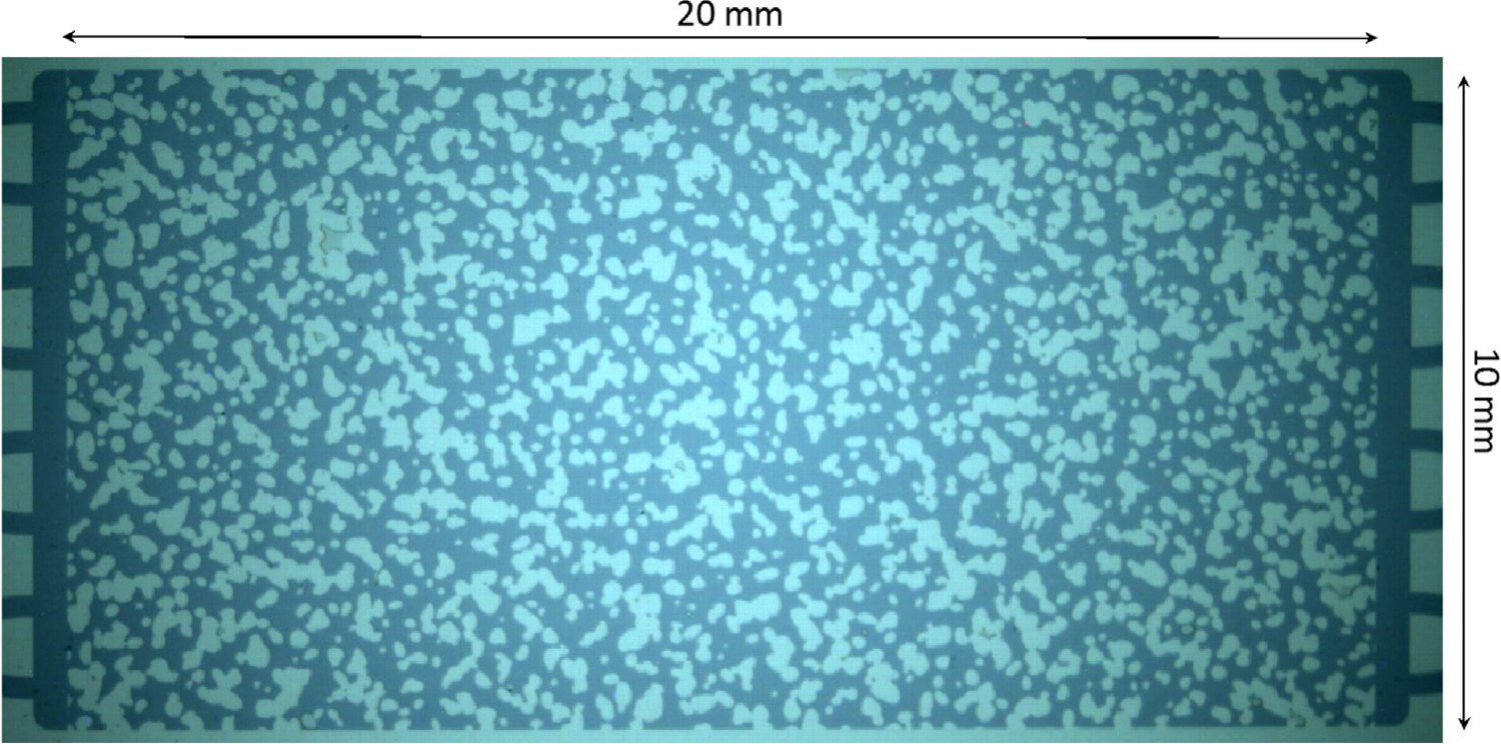
Physical rock network porous media chip (light blue: grain area; and dark blue: pore area).

The permeabilities estimated using the proposed semi-experimental procedure and the Kozeny-Carman equation are in the same order of magnitude and reasonably close. However, it should be noted that the theoretical estimation of permeability, using equations such as Kozeny-Carman’s, for real subsurface porous media microfluidic chips is very difficult (if at all possible) due to complex heterogeneous grain geometries as shown in Fig. 5. Therefore, for such heterogeneous porous media, the proposed method of permeability estimation can be very useful.

## Conclusions

A simple semi-experimental procedure to estimate the permeability of a microfluidic pore network has been proposed. This method does not require any differential pressure sensor or absolute pressure sensors across the porous media for measuring the pressure drop in the porous media. The proposed method also eliminates the possibility of gas entrapment as the method does not require any dead-end tubing perpendicularly connected to the main flow line. The pressure drops in the inlet and outlet channels of the pore network can be significant compared to the pressure drop in the pore network, and hence they can’t be ignored for permeability estimation. An analytical model or a correlation appropriate for the shape of the channel cross section should be used to estimate the pressure drops in the inlet and outlet channels of the pore network. As expected, the estimated permeabilities at flow rates ranging from 50 to 175 μL/min are fairly a constant and the average of the permeabilities is 6.39 ± 0.28 Darcy. The estimated permeability matches reasonably well with the permeability (˜4.87 Darcy) obtained from Kozeny-Carman equation. Another major advantage of the proposed semi-experimental procedure for permeability estimation is its applicability for heterogeneous pore networks for which the Kozeny-Carman equation is not practical.
